# On Mr. Ring's Treatment of Diarrhœa, &c.

**Published:** 1804-09-01

**Authors:** 


### *To the Editors of the Medical and Physical Journal.*

GENTLEMEN,

PERMIT me to observe on Mr. Ring's Paper on Diarrhœa and Cholera Morbus, (p. 102,) that he seems to be unacquainted with the last alterations in the Pharmacopœia Londinensis. The mistura cretacea of the present Dispensatory differs from the mistura e cretâ of the first edition as well as from the julepum e cretâ of the old Dispensatory.

In the julepum e cretâ, *two drachms* of gum arabic were directed for a quart of mixture; in the mistura e cretâ *two ounces;* now, the name is changed to mistura cretacea and a quart of it is to contain *one ounce* of gum arabic. This quantity of gum does not make the mixture too thick; and as the gum is a very useful auxiliary, the alteration is much to be commended.

The plan of treatment which Mr. Ring recommends is not novel, but indisputably useful and practical; and I am persuaded that similar communications on prevailing diseases would be well received by the readers of your Journal.

A very great mortality has lately prevailed among leeches. In the course of three days I lost upwards of twenty; and had my stock amounted to twenty score, I believe they would all in like manner have perished. Another gentleman, I am told, lost one hundred and fifty in a very short space of time, and others have been equally

2 unfortunate.

unfortunate. Though leeches are of very extensive utility in practice, their extravagant price* places them beyond the reach of many patients, and few apothecaries can afford to lose so much money as is incurred by such sudden and numerous deaths; any kind of information therefore which your Correspondents can give upon the best mode of preserving leeches, will be very acceptable to one part at least of the medical world.

Those which I lost were apparently in very good health not a week before they died. They were kept in a sufficiently capacious glass vessel, and were supplied with (Thames) water every day. Almost at once they all sunk to the bottom of the vessel, contracted themselves, and scarcely moving for several hours at last died. Nothing more was particularly observable, except that hard knots or irregular contractions were to be felt in different parts of them.

It ought to be mentioned, that the Thames water has lately been so extremely filthy and offensive to the sight, the smell, and the taste, as to be hardly bearable; but as a similar mortality among leeches occurred during the winter months, when the water was free from such disgusting foulness, I can hardly believe that this could be the cause.

S. M.

*August* 6, 1804.

* In February and March last they could not be purchased in Covent Garden Market for less than 3s. 6d. a piece; at present they fetch eight or nine shillings a dozen. Less than twelve years ago the writer of this paper bought them for 2s. 6d. and 3s. a hundred.

